# Family Systems Intelligence in Couple and Family Therapy—And Beyond

**DOI:** 10.1111/jmft.70136

**Published:** 2026-04-21

**Authors:** William J. Doherty, Adam R. Fisher, Nathan R. Hardy

**Affiliations:** ^1^ Family Social Science University of Minnesota St. Paul Minnesota USA; ^2^ Counseling Psychology and Special Education Brigham Young University Provo Utah USA; ^3^ Family and Consumer Sciences University of Hawai'i at Manoa Manoa Hawaii USA

**Keywords:** couple therapy, emotional intelligence, family systems, medical family therapy, multilateral dynamics, performance‐based assessment, stepfamilies, systemic thinking, systems intelligence, training

## Abstract

This paper introduces family systems intelligence as a capacity that extends beyond emotional intelligence to how individuals understand and respond effectively to family challenges in ways that foster personal and relational health. After defining the general concept of systems intelligence, we define family systems intelligence and distinguish between dyadic and multilateral levels. We argue that couple therapy models are sometimes insufficient when couples face issues involving broader family systems. We illustrate these ideas in three domains where third‐party dynamics are central: stepfamilies, families facing medical illness, and ideological differences. We describe initial work on a performance‐based measure of family systems intelligence, with the goal of a scalable assessment to support research and training. We conclude with implications for strengthening training in systemic competence among therapists and for influencing the broader culture.

This paper introduces the concept of family systems intelligence to the couple and family therapy field, extending ideas developed previously for family science (Doherty 2024). The basic idea is that family systems intelligence goes beyond emotional intelligence to describe a person's ability to understand couple and family interactions as they unfold in everyday life and to respond effectively in ways that foster personal and relational health. Although it builds on foundational theories of family therapy, family systems intelligence is a personal capacity that can be developed in clients rather than an approach to intervention.

After defining the general concept of systems intelligence and the specific idea of family systems intelligence, we go on to distinguish between dyadic and multilateral family systems levels and argue that the latter may sometimes be necessary to understand and treat dyadic relationships. We apply these ideas to stepfamily dynamics and to families dealing with medical illness and ideological differences. Finally, we describe initial work on a measure of systems intelligence for research and training purposes and discuss implications for expanding the goals of systemically oriented therapies to enhance systems intelligence in therapy and the broader culture.

## 1 Emotional Intelligence

In a challenge to the strictly cognitive and unitary conceptualization of intelligence, Gardner (1983) proposed that intelligence involves different types, one of which he termed intrapersonal intelligence, or the ability to understand oneself and one's emotions. Subsequently, Salovey and Mayer (1990) proposed a comprehensive model of emotional intelligence, which they defined as the “ability to monitor one's own and other's feelings and emotions, to discriminate among them, and to use their information to guide one's thinking and actions” (p. 189). In subsequent decades emotional intelligence became a fruitful and widely used concept in research and application in a range of fields (e.g., Chen et al. 2025; Joseph and Newman 2010).

The term emotional intelligence entered the wider culture through Goleman's (1995) bestselling book titled *Emotional Intelligence: Why It Can Matter More Than IQ*. Goleman proposed that emotional intelligence is a critical factor in personal relationships and workplace performance (see also Srivastava 2013). His writing for the lay public was followed by many other authors (e.g., Bradbury 2023). Emotional intelligence was applied to families via books coaching parents on how to develop emotional intelligence in their children (e.g., Tobias and Elias 2000). In all of this literature, the primary focus has been on understanding and regulating one's own emotions and knowing how to read and respond effectively to the emotions of others. As useful as this concept has been for scholars and the public, we believe it is limited by its focus on emotion to the relative exclusion of social/behavioral interactions and social context (Doherty 2024). In terms of family functioning, emotional intelligence is an important building block but not nearly the whole building.

## 2 Conceptual Foundations of Systems Intelligence

Systems thinking was introduced to the therapy world by family therapy pioneers such as Gregory Bateson (1972), Murray Bowen (1976), and Salvador Minuchin (1974). A fundamental insight was that interactions between people take on a life of their own and influence actions and outcomes beyond the characteristics of individuals. Triangular interactions in particular become the building blocks of family dynamics, and changing those dynamics may be necessary for releasing individuals from problematic issues in their lives. With the exception of Bowen, however—whose intervention model emphasized helping clients understand how their family or origin dynamics are playing out now—the foundational models of family therapy focused more on the therapist's knowledge of family systems than on how the clients understood their own systems. The outcomes were improved family and individual functioning, not the family's insights into family systems dynamics. In this paper we build upon the foundation of family systems theories to articulate the implications for personal development in the form of family systems intelligence.

We begin with the general concept of *systems intelligence*, which was introduced into the academic literature by Finnish organizational theorists Saarinen and Hämäläinen (2007) to describe an individual's capacity to understand and act constructively within complex organizations. (The first author of the current paper had been using the term systems intelligence in training therapists prior to discovering the published work of Hämäläinen and Saarinen.) The need for the concept of systems intelligence emerged from a critique of psychology's focus on *emotional intelligence* for being too individualistic and intrapsychic to account for what people need to be effective in complex organizations. Hämäläinen and Saarinen drew on the large literature on systems theory going back to von Bertalanffy (1968), although they showed no familiarity with family systems theory. Their innovation involved formulating systems intelligence not as a theory about systems or a set of professional practices but as a human capacity that can be cultivated to help people be more effective in the systems they are embedded in.

In a similar critique of the limits of emotional intelligence, Doherty (2024) argued that individuals can have a high level of emotional intelligence while lacking a similar degree of insight into the relational systems they are part of. This explains a phenomenon observed by couple therapists: that clients in long‐term individual therapy who have a complex understanding of their inner emotional lives often lack a basic understanding of their own contributions to the relationship patterns that are troubling them. For example, people who have developed intrapsychic insight sometimes take on the role of unwelcome therapist to a partner who usually responds oppositionally, thereby demonstrating that they are not as enlightened as their well‐therapized partner. Emotional intelligence not balanced by interpersonal systems intelligence can distort relationships.

In this paper, we treat *systems intelligence* as an overarching human capacity, and *family systems intelligence* as its application within intimate and family systems. Saarinen and Hämäläinen (2007) offered the following definition of systems intelligence and described how it is manifested:

By systems intelligence, we mean intelligent behavior in the context of complex systems involving interaction and feedback. A subject acting with systems intelligence engages successfully and productively with the holistic feedback mechanisms of her environment. She perceives herself as part of a whole, the influence of the whole upon herself as well as her own influence upon the whole. By observing her own interdependence in the feedback intensive environment, she is able to act intelligently.

(p.1)

Note that the concept involves a cognitive component (understanding systemic dynamics) and a behavioral component (acting effectively within a system).

We believe this general concept of systems intelligence is most useful when applied to specific systems, as scholars have done for business and ecological systems (Törmänen et al. 2022; Sweeney and Sterman 2007). To this point about domain‐specific intelligence, the ability to think and act intelligently within a business or political system would not necessarily carry over to one's family system, and vice versa (although it could be argued that grasping systemic principles in one context could make it easier to learn to apply them in other contexts). Every therapist has treated clients who navigate their work world with insight, nuance, and skill while remaining clueless about the dynamics of their own marriage and family. And in fairness, many therapists with good family systems intelligence would find themselves quickly in over their heads as leaders in a large business organization.

In terms of the family domain, we define family systems intelligence as *the capacity to understand the dynamics of family interactions and to act effectively within those relationships*. (The term family can be applied broadly to close relationships involving history, interdependence, and commitment.) Core assumptions here are that people differ not only in their roles within a family system but also in their capacity to understand and act within that system, and that systemically sophisticated therapy can help people not just solve their immediate relational problems but develop greater family systems intelligence and better manage future challenges. In other words, therapists ideally are not just problem solvers but capacity builders for family systems intelligence. Given that this capacity can be developed over time, therapy can be an important agent of personal development during times of distress.

Note that family systems intelligence has both cognitive and behavioral dimensions: understanding one's family patterns and the ability to act effectively in light of these patterns. This raises the question of whether individuals must be able to conceptualize and articulate complex systems terms. We believe that individuals can have implicit as well as explicit knowledge of family systems. They can have intuitions based on experience without always being able to explain their insights in conceptual terms. This point is consistent with research on the construct of social intuition, which is how people learn about behavioral regularities in their social world without necessarily being able to articulate the basis for their judgments (see Lieberman 2000). Family systems intelligence, then, does not require the use of explicit systemic concepts. As Doherty (2024) illustrated,

The aphorism “blood talks to blood” when it comes to difficult negotiations with in‐laws reflects an understanding of differential attachment and loyalty dynamics. Couples who agree that the one with the original family relationship ought to take the lead for potentially tense conversations with family of origin may be demonstrating systems intelligence without articulating an explicit conceptual explanation.

(p. 736)

Family systems intelligence, then, involves an informed competency for effective action in one's family system—with the “informed” part being either explicit or intuitive. In this light, cognitive understanding alone is not sufficient, just as emotional self‐awareness without self‐regulation is not sufficient for emotional intelligence. On the other hand, just following a therapist's lead, say, in establishing a better boundary between the parental and child subsystems does not necessarily enhance family systems intelligence if the parent does not understand why such a boundary is important and how it was missing before. This example addresses the issue discussed earlier about whether family therapy models help people understand what is going on with their family as part of changing those dynamics.

Next, we introduce a distinction between intelligence related to dyadic systems and multilateral systems in families, a difference with important conceptual and clinical implications.

## 3 Dyadic and Multilateral Family Systems Intelligence

Dyadic systems intelligence (henceforth, *dyadic intelligence*) refers to the capacity to understand and act effectively in couples and other close one‐to‐one relationships. Multilateral family systems intelligence (henceforth, *family‐level intelligence)* encompasses both dyadic relationships and broader dynamics involving three or more family members. While both levels are systemic, they differ in scope.

The field of couple therapy focuses on the emergent properties of dyadic relationships that transcend the psychological dynamics of each individual in the couple relationship. Couple therapists help partners understand how they co‐create patterns such as demand‐withdraw or conflict avoidance, and show how they can collaboratively shift these patterns in healthier directions. Whatever the theoretical orientations behind couple therapy models, they all emphasize the importance of the couple's interactional system that transcends individual psychological dynamics (Lebow and Snyder 2023; Snyder and Lebow 2024). In other words, despite differences in how much they emphasize individual psychological issues as sources of couples' problems, all couple therapy models prepare therapists to assess and intervene with current interaction patterns as a key pathway towards healing. This is in contrast to individually oriented therapists who treat couples mainly in terms of their individual psychological issues, with limited focus on their interpersonal systemic dynamics.

Just as dyadic intelligence is an important leap beyond emotional intelligence, the same is true for family‐level intelligence. We argue that multilateral relationships require a degree of understanding and interpersonal competency beyond what is required for one‐to‐one relationships. This distinction reflects a central insight of systemic family therapy (Bowen 1976; Minuchin 1974), namely, that true systems thinking begins with triads, which involve the complexities of triangles, coalitions, multigenerational boundaries, and competing loyalties. Mathematically, a dyad consists of one bilateral relationship, whereas a triad involves six relationships: three bilateral relationships plus three triangular relationships involving each individual in relationship to the relationship between the other two. For example, a child is in a relationship not just with each parent, but with the parents' relationship. Family members are connected not just to others in a series of dyadic relationships but also to the ongoing relational patterns between family members (in triadic relationships and beyond).

To convey the jump in complexity from dyads to triads, consider a well‐known analogy from astrophysics. As explained by Montgomery (2019), in systems with two interacting celestial bodies, such as a planet and a star, their motions can be predicted with high accuracy using classical physics. But when a third body enters the system, the interactions become exponentially more complex. The so‐called “three‐body problem” refers to the challenge of predicting the movement of three gravitationally interacting bodies, which generates nonlinear, emergent properties that make prediction extremely difficult. The three‐body scenario typically yields only a range of probable outcomes, not a single exact path. This illustrates the special dynamics that arise when a third party interacts meaningfully with a dyadic system. New tools are needed to understand what is going on.

### 3.1 The Limits of Dyadic Intelligence in Clinical Practice

For understandable reasons, most models of couple therapy focus on dyadic communication patterns, attachment dynamics, and problem solving between the two partners who present for therapy (see Lebow and Synder 2023). Couple therapy has paid less attention to the third‐party relationships that may be influencing a couple's functioning—relationships with children, parents, siblings, ex‐spouses, close friends, employers, or other clinical professionals. We propose that managing couple tensions related to these third parties can benefit from a form of intelligence qualitatively different from two‐person relationships.

Stated differently, just as emotional intelligence is not sufficient for navigating complex dyadic interactions, dyadic intelligence—the capacity to co‐regulate, attune, and collaborate with a close other—is often not sufficient when the relationship problem involves broader, multilateral systemic dynamics. One spouse's ongoing conflict with an adult child, for example, can drive wedges in the marital relationship that may require that spouse dealing differently not just with their partner but with that adult child. The same with a jealous ex‐spouse who is trying to undermine the new marriage of their former partner: The dyadic focus of traditional couple therapy might not be sufficient to guide how the therapist handles a situation calling for boundary setting with a third party. Although a competent couple therapist might provide this kind of help, the intervention would have to be guided by a broader framework than a couple therapy model.

To illustrate what family‐level systems intelligence looks like in practice—and what may be missed when therapy stays focused on the dyadic level—we begin with the example of stepfamilies.

### 3.2 Couples in Stepfamilies

Stepfamilies are crucibles of systemic complexity (Bray and Easling 2005; Hetherington, Ganong and Coleman 2017; Papernow 2023). Role ambiguity, loyalty binds, and legacy burdens are common and deeply entangled. A stepparent may be seen both as a threat and a caregiver; siblings may straddle lines between full, half, and step. Communication patterns are often strained by complex histories and intensified by ongoing ambiguity about what roles are acceptable to different family members across generations. These dynamics go far beyond dyadic patterns even when the clinical case presents—as it often does—as a couple problem. And needless to say, the dynamics are far more complex than what emotional intelligence can account for.

In stepfamilies, the biological parent has a prior loyalty and sense of responsibility to their children that is not shared in the same way by the new partner. This can lead to requests for relational priority from a new spouse being experienced as creating a loyalty bind. In one clinical example with a couple, a nagging conflict occurred after they married and moved into the husband's house with his two teenage children. The wife, whose job required her to wake up early in the morning, had trouble going to sleep because of the volume of the teenagers' music. When she asked them to lower the volume because she needed to sleep, they would grumble, lower it for a while, and then crank it up again. When she complained to her husband, he said he understood her need for sleep but the kids needed their music to relax and the volume did not seem excessive. (He avoided conflict with his kids partly because he felt bad about putting them through a difficult divorce.) The clinical intervention here did not derive only from a dyadic couple therapy model—although clearly there was lots to work within the couple dynamic—but also from a multilateral family systems perspective. Namely, the husband needed to assume the role of authoritative parent, redefining his relationship with his children, and in this situation insist that his kids respect his wife's need for sleep. This was the initial clinical intervention, a triangular move that opened the door to further work with the couple.

As Doherty (2024) has pointed out, without family‐level intelligence, the problems of stepfamilies can get reduced to one or more “bad actors” causing problems—most notably, “wicked stepmothers”—or to deficiencies in the couple relationship. We have worked with remarried couples whose only serious problem came in their coparenting of stepchildren, not the rest of their couple relationship. In one case, an otherwise well‐functioning couple included a child psychologist with strong dyadic abilities to relate to teens, but who could not fathom why his new stepchildren would not accept him. It turned out that they resented their mother for divorcing their father and bringing someone new into the household. The table was set for them to remain aloof from a stepparent regardless of the functioning level of the couple and the stepfather's knowledge and skill in relating to teens. Understanding these systemic dynamics, which had not been part of the husband's education as a child psychologist, helped him not personalize the children's reactions and helped both spouses understand how much time it would take to create a new family. They had enhanced their family‐level systems intelligence.

### 3.3 Couples and Families Facing Medical Illness

No couple is an island when a serious illness enters their life. Here we draw on the literature of medical family therapy to argue that effective coping with serious and chronic illness occurs best when family‐level systems intelligence is brought to bear (McDaniel et al. 2014; Rolland 2019; Mendenhall et al. 2018). Illness brings new roles and relationships, some internal, such as caregiver and care recipient, and some external, like patient and medical treatment professionals, all interacting one with one another. The resulting stressors and adaptations cannot be reduced to the emotional lives of the individuals involved or couple or other dyadic relationships in the family. For example, successful coping for a couple may depend on third‐party support from other family members, which brings not just assistance but new relational triangles to be navigated.

When a couple or family needs ongoing medical services for a member, their boundaries have to open to outside professionals who interact with the patient and family members in different ways. This creates opportunities for both supportive and unsupportive interactions that reverberate through couple and family relationships. In one case, the mother felt caught between the pediatrician's caution about an asthmatic, very allergic child being exposed to cat hair at friends' birthday parties and the father's desire to have the child live a normal life. (The couple were in continual disagreement along these lines.) The systemic intervention was to have the father accompany the mother at consultation with the physician, at which the three of them worked out a compromise. This shifted the couple's teamwork and their collaboration with the medical team.

Some family members develop systemic competencies when dealing with helping professionals and health care organizations, while others become confused and frustrated. Emphasizing family‐level intelligence as a capacity that can be applied to internal dynamics and also to interactions with medical and other systems opens the door to therapeutic interventions well‐developed by medical family therapists (McDaniel et al. 2014). As with clients in stepfamilies, focusing on emotional intelligence or dyadic intelligence alone would not be sufficient for optimal treatment.

### 3.4 Ideological Differences in Families

Ideological commitments are embedded in families, communities, and institutions. From religious affiliation (e.g., mixed‐faith marriages) to political views (e.g., Democrat–Republican couples or adult siblings in the United States), these differences often involve third‐party involvement and concerns. For instance, adolescent and adult children may be expected to take sides in their parents' differences over religion or politics. Clergy or members of a tight‐knit religious congregation may express their attitudes about the non‐participating spouse. Adding a layer of emotional complexity, some partners feel a sense of betrayal when these differences emerge after they marry, such as one partner changing religious beliefs and affiliation. The whole family's moral and relational structure is now being reorganized. These changes can introduce new systemic complexity in a variety of areas, such as parenting: Which ideology will be taught to the children? How will the members of the family navigate coalitions around these ideologies? How will triangles be addressed when they involve grandparents?

Major models of couple therapy might frame this kind of ideological conflict in normative terms—for example, as “perpetual problems” in Gottman method (Gottman and Gottman 2024), or simply as “differences” in integrative behavioral couple therapy's DEEP analysis (Christensen et al. 2020). In either case, the problem is framed dyadically. We argue that this issue is often closely tied to larger family and community dynamics, with feelings of loyalty and betrayal involving these third parties.

When we work with couples where one partner made a change in religious affiliation, we try to move from an individual lens focused primarily on feelings, through a dyadic “mixed‐faith marital conflict,” to a more systemic “reorganization” of the family. We encourage both parents to avoid allying with their children and to agree as a couple in terms of how to handle disagreements that happen in front of their kids. Couples can acknowledge that their children may align with each parent at different stages, and avoid the temptation of intensifying their beliefs in the face of relational anxiety in the family. The point here is that the family systems level of understanding, because it adds dynamics such as multigenerational boundaries, differentiation of self, and triangulation, can enhance the treatment of ideologically divided couples.

### 3.5 Measurement

Future development of the concept of family systems intelligence involves assessment for research and training purposes. Here we describe our initial approach to this task (we plan future reports after we have tested a measure). Fortunately, there is an extensive literature on measuring emotional intelligence that can be drawn on (see review by O'Connor et al. 2019). A key distinction in that literature is between emotional competency via self‐report (how well someone believes they deal with emotion) versus performance testing (usually assessed by the level of emotional complexity someone shows when analyzing vignettes of people in challenging emotional situations). For our purposes, performance measurement is preferable because of concerns about the accuracy of self‐reports of systemic understanding.

In addition, we want to develop a measure that can be reliably scored with AI assistance so that it can be used by clinicians and not just researchers who go through extensive coding training. (See Elyoseph et al. 2023, for a successful example of reliable coding by ChatGPT of a performance measure of emotional awareness.) Finally, we envision a measure that can be used to assess the family systems intelligence of therapists as well as lay people, so that it can be used for training purposes.

Our team initially constructed 16 vignettes describing conflicts involving three or more people in a family system. These scenarios include post‐divorce conflict, in‐laws, stepfamilies, interactions with the medical system, family cutoffs, and holiday traditions. Each vignette presents the scenario and then asks the respondent: “1. What do you see as key contributors to the problems in this situation?” and “2. In your opinion, what would need to happen to address the situation?” Directions ask to not just suggest outside help (like counseling) as we are looking for participants' own ideas.

Responses to the two questions for each vignette are then coded based on the level of systemic complexity (ranging from no analysis at all, to individual level, to dyadic level, to multilateral level). Of note, scoring is not based on a particular model of family systems theory but on the level of complexity of the responses. We are currently developing a codebook and analyzing pilot data from lay people and therapists. At some point we will train ChatGPT with the codebook and sample cases, using an iterative process that prior research suggests can yield reliable scoring and greatly expand the utility of the measure (Elyoseph et al. 2023).

### 3.6 Training

Recent reviews of couple and family intervention work point to the field's need to address complex multisystemic issues and to expand development and training to match that complexity (Snyder and Balderrama‐Durbin 2020; Stanton and Welsh 2012). From our perspective, this means helping trainees move fluidly between individual, dyadic, and family‐level intelligence, with the biggest current gap being at the family systems level described in this paper. The largest current gap is not trainees' ability to name systemic concepts, but their ability to *operationalize* multilateral thinking when real third parties (children, ex‐partners, extended kin, schools, medical teams, congregations, workplaces) are exerting pressure on the focal relationship system. Kaslow et al. (2019) summarize this kind of training stance as follows:

Consistent with the assertions of other educators and trainers, we concur that it is crucial for trainees and professionals to think and act like a couple and family psychologist…. Regardless of one's theoretical orientation, this requires one to develop the capacity to think systemically in accord with general systems theory. Systemic thinking offers both a framework and a lexicon for describing and understanding the factors and interrelationships that inform the adaptive and dysfunctional behavior of systems, and affords us a road map for being attuned to biopsychosocial factors that influence social/relational contexts and systems…. Learning to think systemically typically necessitates an epistemological shift in the way one sees the world and understands human behavior away from an individualistic psychological perspective to a contextual conceptualization of human behavior and interactions.

(p. 4)

Note that this call for training improvement does not make the distinction between dyadic systems and multilateral systems. In practical terms, we suggest that training targets at least four teachable competencies that would advance the field beyond its current focus dyadic systems: (1) systems assessment—identifying the relevant systems, their key members, and the active triangles, coalitions, and boundary processes; (2) systemic case formulation—linking the couple's presenting cycle to multilateral patterns across contexts; (3) multilateral intervention—coordinating boundary negotiations and structured contacts with third parties when appropriate (e.g., co‐parenting partners, extended kin, schools, or medical teams), while maintaining a clear couple‐therapy frame; and (4) therapist‐as‐systemic actor—monitoring one's own positioning, reactivity, and alliance patterns in the face of multilateral pressures. These skills can be developed through structured exercises that ask trainees to build a systems map alongside a dyadic formulation, identify leverage points for shifting multilateral patterns, and practice brief role‐plays that include third‐party dilemmas (e.g., a co‐parenting conflict with an ex‐partner, a stepfamily loyalty bind, or a consult call with a physician).

Supervision is a natural home for this work because supervision itself is a multilateral system (clients, trainee, supervisor). Video review can attend not only to dyadic process but to how the therapist tracks and responds to triangles, boundary moves, and alliance shifts as they emerge. Competence can be evaluated with case‐based rubrics, structured case presentations that require an explicit multilateral hypothesis, or simulation encounters that require trainees to demonstrate systemic tracking and select interventions that are both effective and ethically and relationally responsible.

Taken together, a training emphasis on systems intelligence can help the field retain its strengths in dyadic process while becoming more agile in the multilateral realities that couples bring to therapy.

## 4 Broader Implications of Family Systems Intelligence

Next, we offer three potential implications of the ideas presented in this paper, ranging from the clinical system to broader societal and cultural systems.

### 4.1 Systems Intelligence as an Outcome of Couple and Family Therapy

Just as some prominent forms of individual psychotherapy (including psychodynamic and humanistic) can be viewed as implicitly enhancing emotional intelligence as a capacity beyond the specific goals of treatment, we suggest that an outcome of couple and family therapy might be the enhancing of family systems intelligence in addition to solving immediate relationship problems. We believe that this increased capacity of clients to understand and act effectively in facing future challenges—beyond those dealt with in therapy—is already an implicit outcome of many forms of couple and family therapy. In the couple therapy area, emotionally focused, Gottman method, and integrative behavioral couple therapy, despite their many differences, all help couples identify and track their problematic interaction patterns, a capability that presumably has payoffs for couple coping in the future. Stated differently, greater dyadic systems intelligence can be viewed as resilience outcome of successful couple therapy. To be clear, we are not proposing that all couple therapy models should have a stated goal of enhancing dyadic systems intelligence, but we are suggesting this is an indirect outcome of successful couple therapy for many couples.

Family therapy models present more ambiguity for promoting family systems intelligence. Many of the classic models (structural, strategic, Palo Alto) focused on direct therapist intervention with current problems, with less emphasis on family members understanding their dynamics. Bowen's model offers the strongest case for enhancing family systems intelligence because of its focus on investigating intergenerational family patterns and learning to differentiate from fused relationships and unhealthy triangles. Beyond the classic models, contemporary adaptations such as the medical family therapy and stepfamily therapy models discussed in this paper are more explicit in helping clients understand their internal dynamic—and the case of medical family therapy, their interactions with medical provider systems—and thus improve their capacity to cope with challenges more effectively when therapy has ended and further life stresses emerge. For example, clients might be able to identify an interaction pattern such as overfunctioning and underfunctioning roles that they tend to fall into when under external stressors and articulate how they would resist that pattern in practice.

What might be a practical benefit of viewing greater family systems intelligence as a desirable outcome of couple and family therapy—and, we would argue, of individual therapy that focuses on couple and family problems? It could focus more attention on what we see as the major benefit of family systems intelligence, namely, enhancing resilience or the capacity to adapt and solve emerging problems in the future. This would be a testable hypothesis if outcome studies examined not just traditional functional outcomes but also clients' understanding of their systems dynamics—and then whether such understanding predicted successful long‐term coping. For example, clients could be assessed for their understanding of the systemic dynamics that changed as a result of therapy, and for views of how they would like to handle future stresses and challenges they face as a couple. Another measurement possibility would be the tool we are developing (described above) that measures overall family systems intelligence as applied to vignettes about other families' experiences. The key would be whether such indicators of family systems intelligence are associated with better short term and long‐term functioning outcomes.

### 4.2 Cultural Awareness of Family Systems Intelligence

Another potential implication of the family systems intelligence concept is to spread systemic ideas and practices throughout society. There is widespread consensus among historians and sociologists about the cultural influence of individual therapy models, perhaps because they fit well with individualist Western culture (Aubrey and Travis 2015; Bellah et al. 1985; Rieff 1987). But systemically informed therapies appear to have had far less impact at the cultural level. As a society we teach children the language of emotion but not the language of systems. Books, podcasts, and social media reels offer a plethora of psychological analysis along with basic relational terms such as “communication problems” but not much in the way of systemic ideas to understand relational problems. For example, there is relatively little use of notions such as dyadic and family interaction patterns, strategies for perspective taking and de‐escalation during conflict, and the role of triangles and coalitions in chronic relational problems. An example is the understanding of adaptation to remarriage and stepfamily life, where ungrateful stepchildren and wicked stepmother explanations for problems are culturally sticky. Unfortunately, we believe the same understanding gap is often true in individual therapy, as documented in a study of client reports of how often their individual therapists focused on the psychopathology of the relationship partner whom the therapist had not met (Doherty and Harris 2022). We believe that systems‐informed individual therapists can help clients better understand how they are caught up in troublesome couple interaction patterns or facing confusing triangulation with their families, and take more effective action to change their participation in these patterns.

We are not the first systems‐oriented therapists to call for more systems understanding in the broader culture (Bateson 1972; Bowen 1976; Minuchin et al. 1967). However, a strategic advantage of using the term “intelligence” is that it frames systems thinking and action as a personal capacity (understanding and behavioral effectiveness) that can be developed, not just as a theoretical lens that is useful in therapy. Just as gaining emotional intelligence has become a valued goal in the broader culture (as witnessed by the proliferation of self‐help books and podcasts), could becoming more intelligent about family systems become equally popular and valued? It appeals to the traditional American focus on personal development and suggests that family systems intelligence is a capacity worth pursuing because it contributes to a flourishing life. At the level of cultural framing, just as emotional intelligence has been termed *EQ* in popular writing (e.g., Stein and Book 2011) and in academic literature when applied to measurement (Dawda and Hart 2000), perhaps referring to family systems intelligence as *SQ* and dyadic intelligence as *RQ* (for relational intelligence) could enhance their appeal. Whether or not such labels are adopted and prove useful, the core point is that understanding and effective functioning in systems can be framed as a form of practical intelligence that people can aspire to develop throughout their lives.

### 4.3 Application to Societal Problems

As mentioned, family therapy pioneers had aspirations for their ideas to make a difference at the societal level. The concept of family systems intelligence offers a new possibility for this kind of broader impact by equipping people with the knowledge and skills for understanding and addressing problems affecting society. We offer the example of the application of couple therapy principles and practices in the work of the national nonprofit Braver Angels on political polarization of “reds” and “blues” in the United States (Doherty and Mendenhall 2024; see also Laszloffy and Platt 2024). The workshop model of Braver Angels and in particular the design and activities of its Red/Blue workshop, uses the following central elements of couple therapy articulated by Benson et al. (2012) in their chapter about common mechanisms of change across models of couple therapy.

- Altering views of the relationship away from one‐sided assignment of responsibility.
- Modifying dysfunctional interactional behavior (particularly toxic conflict)
- Eliciting avoided private behavior (such as sharing of vulnerability and affection)
- Improving communication
- Promoting strengths (p. 25)

During this experiential workshop based on these principles, participants come to see how both sides contribute to the problem of polarization, they learn to listen rather than immediately react in the face of opinions that disturb them, they practice self‐criticism of their own side, they learn to frame their viewpoints and questions in a way others might be receptive to, and they articulate the strengths of their own side while listening to how other see the strengths of their political stance. A description of the specific activities that elicit these reactions is contained in Baron et al. (2025). A randomized controlled trial by external researchers found that this workshop reduced polarized and stereotyped attitudes among participants and influenced at least short‐term behavior in a positive direction (Baron et al. 2025). Although the Braver Angels framework primarily uses dyadic systems concepts from couples therapy, one of the key workshops addresses how people who improve their relations with the political “other” can learn to handle the pushback from peers who see them as disloyal—thus, a triangular perspective on working on depolarization.

Of course, it is important to be modest about how ideas from couple and family therapy and family systems intelligence can change the world. More important is how a broader systemic lens can help therapists assist clients when their family interactions are influenced by large systemic forces such as polarization. Therapists can focus on broader social issues when those impinge directly on the personal and relational lives of clients—as is happening increasingly with political polarization (McCown and Chamberlain 2025). And sometimes therapists can venture with humility to apply what we know to the broader problems themselves.

## 5 Conclusion

When introducing a new concept, it is important to be clear about what it is not (the boundaries and limits of the concept) and to outline next steps in its development.

### 5.1 What Family Systems Intelligence Is Not

First, family systems intelligence is not about theoretical knowledge in the way that family systems theory has functioned in our field; it is about practical knowledge about how people understand their couple and family relationships and how they can function effectively in those relationship systems. In the same way, emotional intelligence is not just emotional self‐awareness but also the ability to emotionally self‐regulate and respond to the emotions of others. Family systems intelligence is more akin to wisdom for dealing with relational complexity than it is about conceptual knowledge. People do not have to verbalize family systems theory 101 to show family systems intelligence.

Second, family systems intelligence is not a fixed trait of individuals who are high or low throughout their adult lives, but a developmental capacity that can grow from childhood throughout adulthood and can expand to include new family forms and life stages a person encounters. A trap to be avoided is the creation of a new fixed hierarchy of competence. People might have different levels of systemic intelligence at different times in their life and in different family forms they encounter. As mentioned, therapy might enhance their family systems intelligence as they learn to manage a family crisis.

Third, family systems intelligence is not culture free; family complexity is manifested in different social environments with their own cultural norms for family relations. We suggest that family systems intelligence has some universal dimensions, such as the capacity to deal with multigenerational family relationships, and some culture‐specific dimensions such functioning within traditional versus egalitarian marriage norms and with family ties to different kinds of external systems. What might be effective management of a family crisis in one culture may play poorly in another. However, people in each culture presumably vary in their ability to understand with complexity what is going on and how to cope with it—in other words, their family systems intelligence as played out in their own context. For example, in non‐Western cultures that emphasize in‐law relationships, the specific understanding and effective practices for functioning well as a family member might be different in important ways from those of more nuclear family cultures. The key is how well people assess and act on the complexities of whatever cultural norms they inhabit. How well do they navigate differential authorities, loyalties, and obligations that go deeper than individual personalities or dyadic bonds? That is where family systems intelligence lies.

Fourth, the concept does not suggest that any one model's ideas about relational systems is the gold standard for family systems intelligence. In other words, it is not marked by the use of specific terminology but by the *complexity* of a person's framing of couple and family issues, in particular whether the person frames understanding and action in strictly individual terms or also dyadic, triadic, and beyond. Specific systemic concepts such as behavioral interaction patterns versus attachment dynamics, boundary issues versus multigenerational patterning, are not essential to family systems intelligence; rather, it's the use of dyadic‐level and family‐level ideas and practices, regardless of their pedigree in therapy models. The same is true for how therapists practice: they can be systems‐informed therapists even if their theoretical and practice models are quite different.

Fifth, family systems intelligence does not stand alone and apart as a form of knowledge and effective behavior. It builds on emotional intelligence and would be impossible without it (for an extended discussion of this point, see Doherty 2024). And both of these forms of intelligence are influenced by biological, societal, and cultural forces beyond their immediate scope. Family systems intelligence is not the answer to complex human problems; it's a kind of human capacity for problem solving that has not been given enough notice.

Finally, even though we have made a conceptual and clinical case for the distinction between dyadic systems and family‐level systems intelligence, we do not want to imply that couples therapists using dyadic models cannot be effective with cases involving third party influences. Some couples may figure out on their own how to handle their larger systemic problems once they deal with their dyadic issues. Or the couple therapist might effectively use concepts and tools beyond those in their dyadic model. (Similarly, we believe that it is possible to promote family systems intelligence in individual therapy.) However, we believe that therapists would benefit from explicit family‐level case conceptualizations and interventions when working with individuals and couples who have significant influences from their larger family system.

## 6 Future Directions

Of particular interest for the future would be exploring how family systems intelligence develops from childhood through adulthood. Perhaps emotional intelligence comes first in the early stages of life, and then family systems intelligence develops as individuals become more aware of relational complexities. A related question is how it might it be taught directly versus only through guidance during life transitions and events.

We hope to learn about how well therapists see dyadic and family‐level interaction patterns in their clients' lives, and how often they reduce these to the characteristics of individuals. This connects to the measurement tool we are developing, which we plan to use with both therapists and lay people. It also connects to training where we want to learn how well students can integrate systems ideas and practices into their current models of therapy. And then there are the crucial so‐what questions for therapy: Does it make a difference for outcomes if the therapist uses family‐level systems concepts and tools—and for which kind of cases? Does increasing family systems intelligence to enhance resilience make a difference for long‐term outcomes? These are researchable questions.

Beyond the clinical realm, we want to explore whether the idea of a distinctive human capacity to understand and act effectively in one's intimate world could have cultural resonance in an individualistic culture focused on personal development but lacking systemic subtleties. And could it inform useful social interventions such as seen with Braver Angels? This larger goal reflects the aspirations of the pioneers of systemic therapies: to influence society with the realization that we co‐create our social worlds and can change them if we act together.
